# The Treatment of Gonorrhœa in the Male

**Published:** 1910-03

**Authors:** 


### Reviews of Books.

**The Treatment of Gonorrhœa in the Male.** By CHARLES LEEDHAM-GREEN, M.B. Second Edition. Pp. xii, 160. London: Baillière, Tindall & Cox. 1908.

Within moderate compass this book gives the reader a good and eminently practical account of the treatment of gonorrhœa in the male, and of the methods by which the seat and character of the urethral inflammation are determined.

The newest matter in the second edition of the book is a short description of Goldschmidt's irrigation urethroscope. Whether this ingenious instrument, which gives apparently a much better view of the posterior urethra than that obtainable with ordinary urethroscopes, will prove of much practical value in diagnosis and treatment remains for future decision. The description of the use of ordinary urethroscopes is meagre and will not greatly help the student, who must turn to the larger works of continental writers, especially to the classical works of Oberländer and Kollmann, for detailed information. But the art of using urethroscopes and the ability to correctly interpret the visual impressions obtained with them, and the appreciation of the practical bearings of these, can only be acquired by prolonged and patient practice, not from books. For this reason but few general hospital surgeons, not to mention general practitioners, can use urethroscopes advantageously. Those who wish to do so should first attend special clinics where the instruments are extensively used. Fortunately in the majority of cases of chronic gonorrhœa less difficult methods of examination enable treatment to be successfully conducted.

Much more may be said for the use of the urethral dilators of Oberländer and Kollmann, some varieties of which are described and illustrated in the text. It is surprising and regrettable that these instruments are so little used by British surgeons.

The later part of the book deals briefly with the commoner complications of gonorrhœa, and an important section on the proof of the cure of the disease and its bearing on marriage brings to a conclusion a work which we can cordially recommend to all practitioners, and especially to those who have not the opportunity of studying the more exhaustive treatises on the subject.

